# Multidimensional Gene Regulatory Landscape of Motor Organ Pulvinus in the Model Legume *Medicago truncatula*

**DOI:** 10.3390/ijms23084439

**Published:** 2022-04-18

**Authors:** Quanzi Bai, Wenjing Yang, Guochen Qin, Baolin Zhao, Liangliang He, Xuan Zhang, Weiyue Zhao, Dian Zhou, Ye Liu, Yu Liu, Hua He, Million Tadege, Yan Xiong, Changning Liu, Jianghua Chen

**Affiliations:** 1 CAS Key Laboratory of Topical Plant Resources and Sustainable Use, CAS Center for Excellence in Molecular Plant Sciences, Xishuangbanna Tropical Botanical Garden, Chinese Academy of Sciences, 88 Xuefu Road, Kunming 650223, China; baiquanzi@xtbg.ac.cn (Q.B.); yangwenjing@xtbg.ac.cn (W.Y.); zhaobaolin@xtbg.ac.cn (B.Z.); heliangliang@xtbg.ac.cn (L.H.); zhangxuan@xtbg.ac.cn (X.Z.); zhaoweiyue@xtbg.ac.cn (W.Z.); zd134@mail.ustc.edu.cn (D.Z.); 18256966882@163.com (Y.L.); oklahomaliu@163.com (Y.L.); hehua554@126.com (H.H.); 2 University of Chinese Academy of Sciences, Beijing 100049, China; 3 Shanghai Center for Plant Stress Biology, CAS Center for Excellence in Molecular Plant Sciences, Chinese Academy of Sciences, Shanghai 201602, China; qinguochen@sibs.ac.cn; 4 School of Life Sciences, University of Science and Technology of China, Hefei 230027, China; 5 Department of Plant and Soil Sciences, Institute for Agricultural Biosciences, Oklahoma State University, 3210 Sam Noble Parkway, Ardmore, OK 73401, USA; million.tadege@okstate.edu; 6 Basic Forestry and Proteomics Research Centre, Haixia Institute of Science and Technology, Fujian Agriculture and Forestry University, Fuzhou 350002, China; yanxiong@fafu.edu.cn

**Keywords:** leaf movement, pulvinus, transcriptome, proteome, ELP1, *Medicago truncatula*

## Abstract

Nyctinastic leaf movement of Fabaceae is driven by the tiny motor organ pulvinus located at the base of the leaf or leaflet. Despite the increased understanding of the essential role of *ELONGATED PETIOLULE1* (*ELP1*)/*PETIOLE LIKE PULVINUS* (*PLP*) orthologs in determining pulvinus identity in legumes, key regulatory components and molecular mechanisms underlying this movement remain largely unclear. Here, we used WT pulvinus and the equivalent tissue in the *elp1* mutant to carry out transcriptome and proteome experiments. The omics data indicated that there are multiple cell biological processes altered at the gene expression and protein abundance level during the pulvinus development. In addition, comparative analysis of different leaf tissues provided clues to illuminate the possible common primordium between pulvinus and petiole, as well as the function of ELP1. Furthermore, the auxin pathway, cell wall composition and chloroplast distribution were altered in *elp1* mutants, verifying their important roles in pulvinus development. This study provides a comprehensive insight into the motor organ of the model legume *Medicago truncatula* and further supplies a rich dataset to facilitate the identification of novel players involved in nyctinastic movement.

## 1. Introduction

Plants as sessile organisms have evolved multiple movement behaviors to optimize their development and environmental adaptation. The nyctinastic leaf movement is a rhythmic plant behavior where leaves open during the day and fold at night [1,2,3]. This kind of sleep movement, observed predominantly in species of Leguminosae and Oxalidaceae families, is controlled by the motor organ pulvinus located at the base of the leaf or leaflet, and it has intrigued phytologists since the era of Darwin [1]. Previous studies indicate that the pulvinus consists of a central vascular bundle surrounded by layers of motor cells, and the nyctinastic leaf movement depends on the asymmetric volume change in motor cells in opposite parts of the pulvinus. In recent decades, physiological evidence further supported that transmembrane ion transport and aquaporin channel proteins coordinate with upstream signals, including light and the circadian clock to rebalance the osmotic pressure of motor cells to a swelling or shrinking state [2,3,4,5,6,7,8,9,10]. Recently, the expression of *SLAC1 HOMOLOGUE 1* (*SsSLAH1)* was found to display morning-phased circadian rhythms, and the folding process of leaf in *GmSLAH1*-silenced plants was found to be more gradual [7,11]. However, the molecular mechanism underlying the signaling transduction pathway [3,12] in pulvinus cells is still poorly understood.

A defection of pulvinus development would result in abnormal leaf movement. Particularly, previous research found that ELP1/PLP is a transcription factor belonging to the LBD family and especially expressed in the pulvinus. The mutation of *ELP1* and its orthologs *APULVINIC (APU)* and *SLEEPLESS (SLP)* in *Medicago truncatula, Pisum sativum* and *Lotus japonicas,* respectively, exhibit petiole or rachis-like structures at the pulvinus-equivalent position and a total loss of nyctinastic leaf movement [13,14], revealing a conserved molecular mechanism on motor organ identity determination in related legumes [13,15,16]. Loss-of-function of *Glycine max Increased Leaf Petiole Angle1 (GmILPA1)*, a gene encoding an APC8-like protein, displays a smaller pulvinus, thinner cortex, and fewer and smaller motor cells coming with the abnormal nyctinastic leaf movement [17]. A recent study suggested that the F-box protein SMALL LEAF AND BUSHY1 (SLB1)/MINI ORGAN1 (MIO1) plays a key role in regulating pulvinus development and organ size in *M. truncatula* [18]. In addition, the mutation of *MtDWARF4* was found to regulate the geometry of the compound leaf and subsequently influence nyctinastic leaf movement, revealing that the BR synthesis and signal transduction factors are also involved in the normal function of the pulvinus [19,20]. Although these factors have been identified to participate in pulvinus development, ELP1 is identified as the only key transcription factor involved in pulvinus development, and the underlying molecular mechanism remains unclear. Furthermore, how the special motor organ, which does not exist in most plant families, is initiated in legume plants is still unknown. To elucidate the gene regulatory landscape underlying pulvinus development and signal transduction, the integration of multi-omics analysis of pulvinus is an efficient approach. Although *M. truncatula* is a model species of legumes, its gene expression atlas deriving from publicly available Affymetrix GeneChip data only covers major organ systems without the tiny motor organ pulvinus [21,22]. Until now, no public transcriptome or proteome data on *M. truncatula* pulvinus has been reported. A comprehensive knowledge of the gene regulatory landscape in pulvinus will be very helpful to dissect its signal transduction and development process. In addition, comparative transcriptome analysis of pulvinus and other tissues in compound leaf will facilitate illuminating the origin of the pulvinus in legume plants.

In this study, we simultaneously profiled transcriptome and proteome analysis, and the results elucidated that ELP1 modulates mRNA expression and protein abundance, which were probably related to pulvinus development and signal transduction. In addition, comparison analysis of pulvinus-specific gene expression in compound leaf provided deeper understanding of the gene regulatory landscape and ELP1 function in pulvinus. Meanwhile, the experimental evidence further verified the powerful value of these omics data. This study provides a rich resource to uncover the multiple layers of the gene regulatory network involved in pulvinus development and signal transduction in the model legume *M. truncatula*, further facilitating the investigation of ELP1 function in the regulation of leaf movement.

## 2. Results

### 2.1. Collection of WT Pulvini and the Defecitve Pulvinus of elp1 Mutant for Transcriptome and Proteome Analyses

**Figure 1:**
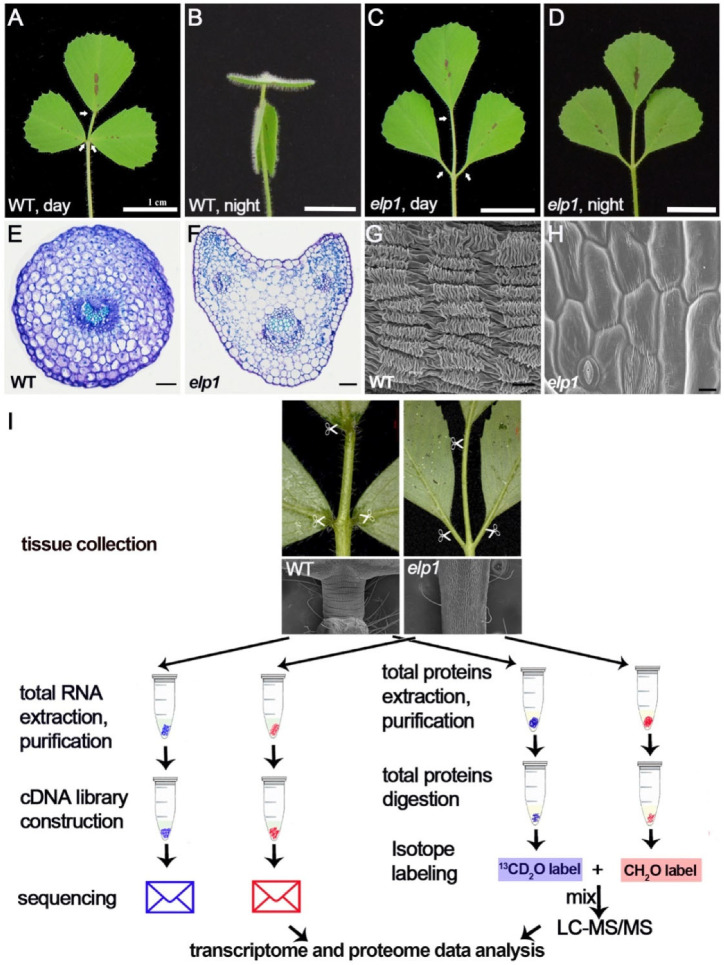
Collection of WT pulvini and the defective pulvinus of *elp1* mutant for transcriptome and proteome analyses. (**A**,**B**) Morphological analysis of 4-week-old seedling of WT. Leaves remain open in horizontal state during the day (**A**) and fold up to vertical position at night (**B**). Bar = 1 cm. (**C**,**D**) Morphological analyses of 4-week-old seedling of *elp1* mutant. Leaves stay open at horizontal level during the day (**C**) and at night (**D**). Bar = 1 cm. Arrows indicate WT pulvini in A and the equivalent positions of *elp1* in C. (**E**–**H**) Histological (**E**,**F**) and morphological (**G**,**H**) analyses between WT pulvinus (**E**,**G**) and *elp1* equivalent tissue (**F**,**H**). Scale bars: (**E**,**F**) 50 µm, (**G**,**H**) 10 µm. (**I**) Schematic workflow of transcriptome and proteome analysis of *elp1* and WT. Collection of WT pulvinus and the defective pulvinus of *elp1* from eight-week seedlings for further multidimensional analyses.

The model legume *Medicago truncatula* displays the nyctinastic leaf movement with the leaf open horizontally in the daytime and closed in an upright position at night (Figure 1A,B). In contrast, the leaves of *elp1-1* mutants remain opened all the time (Figure 1C,D). Phenotype analysis indicated that the motor organ pulvini of the wild type (cv Jemalong A17) are located at the base of leaflets (Figure 1A), while pulvini are totally absent in *elp1-1* mutants (Figure 1C), which are associated with the loss of movement phenotype. Histological and morphological analysis showed a significant difference in the transversal structure, vascular bundle and epidemical cell identity between WT pulvinus and *elp1-1* mutant equivalent tissue (Figure 1E–H).

To understand the underlying molecular mechanism of how ELP1 regulates pulvinus development as well as obtain a comprehensive understanding of signal transduction [3,12] in pulvinus cells, we performed transcriptome and quantitative proteomes analysis using the pulvini of WT (A17) and the tissues from the equivalent position (the defective pulvinus) of the *elp1-1* mutant at the 8-week stage (Figure 1I). To obtain stable and reproducible data, three biological replicates were performed both in the transcriptome and quantitative proteome analyses.

### 2.2. Multiple DEGs Are Involved in Pulvinus Development and Signal Transduction

Principle component analysis (PCA) and heatmap analysis of transcriptional data exhibited apparent separation among these two types of samples, indicating their significant differences (Supplementary Materials Figure S1). Transcript statistics showed that a total of 29,510 genes were detected, which covered around 58% of the Mt4.0 annotation genes [21]. Further analysis identified 244 novel gene loci that have not been annotated in Mt4.0 annotation genes. Collinearity analysis with the Mt5.0 reference genome database indicated 197 of those genes were annotated, while 47 genes were still missing (Supplementary Materials Table S1). To clarify whether these 47 genes were conserved among the different species, comparative transcript analysis among the *Medicago truncatula*, *Cicer arietinum*, *Glycine max, Arabidopsis thatiala, Solanum lycopersicum* and *Oryza sativa* species was conducted, and the result indicated that 36 of 47 novel genes were identified only from *Medicago truncatula* (Supplementary Materials Figure S2). These novel genes not only complement the gene annotation database but also provide a valuable list of candidate genes for the reverse genetic study of pulvinus function in the future.

**Figure 2:**
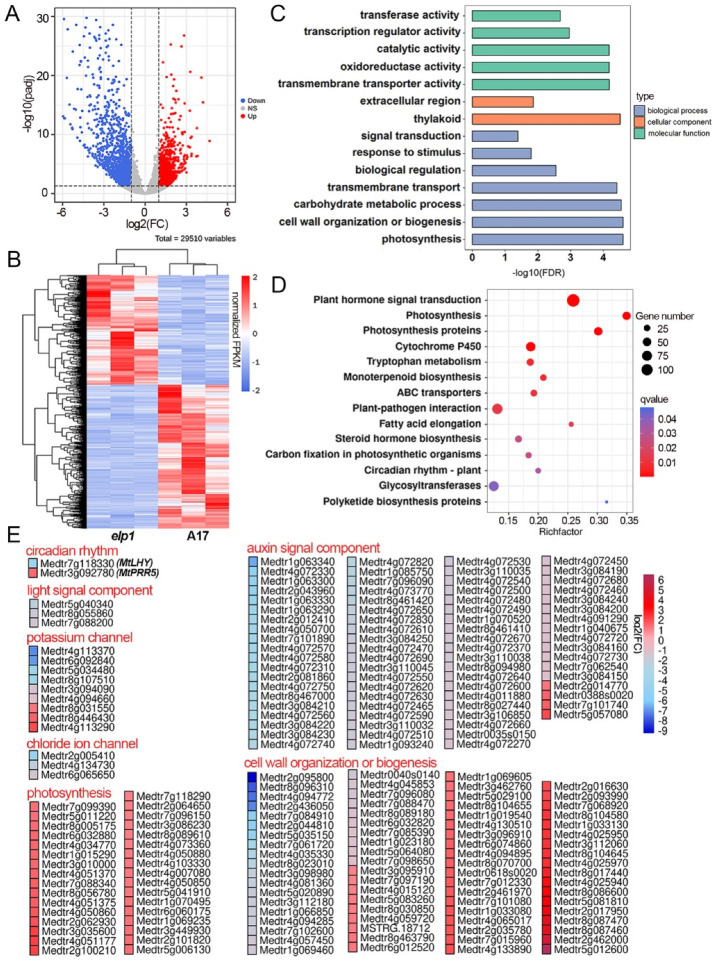
DEGs involved in pulvinus development and signal transduction were identified in *elp1* mutant. (**A**) Volcano plots of differentially expressed genes between WT and *elp1* mutants after RNA-seq analysis. Up-regulated and down-regulated genes are presented in red and blue color dots, respectively. The x-axis represents the natural logarithm of fold change (Fc) and the y-axis represents log_10_ of the adjusted *p*-value of each transcript. The blue and red dots represent the downregulated and upregulated genes, respectively. (**B**) Hierarchical clustering analysis of all the differentially expressed genes (DEGs) in WT (A17) and *elp1* mutant samples. (**C**) Gene ontology (GO) enrichment analysis of the DEGs between WT and elp1 mutants. (**D**) Pathway enrichment of differentially expressed genes. The upregulated DEGs are enriched above blue dotted line, while downregulated DEGs are enriched below the blue dotted line. (**E**) Selected significantly differentially expressed genes in potassium channel, chloride ion channel, circadian rhythm, light signal component, cell wall organization or biogenesis and auxin signal component.

With a threshold of │Log_2_ FC│ ≥ 1 and *p*-adjust value < 0.05 in three biological repeats, a total of 2652 differentially expressed genes (DEGs) with 1506 downregulated and 1146 upregulated candidates were identified in *elp1-1* mutants compared to the wild type (Figure 2A, Supplementary Materials Table S2). Hierarchical clustering analysis of all the DEGs in WT and *elp1-1* mutant samples displayed significant differences between WT and *elp1* mutants (Figure 2B).

In order to address which kinds of gene function or biological pathway were involved in pulvinus development and signal transduction among these 2652 DEGs, we simultaneously carried out gene description and GO terms as well as KEGG pathway analysis. We found that the top GO terms were involved in photosynthesis, cell wall organization or biogenesis, transmembrane transport, transmembrane transporter activity and transcription regulator activity (Figure 2C, Supplementary Materials Table S3). Kyoto Encyclopedia of Genes and Genomes (KEGG) analysis found that plant hormone signal transduction, photosynthesis and circadian rhythm were the significantly changed pathways (Figure 2D, Supplementary Materials Table S4). 

Among the 2652 DEGs, biological clock core regulator *MtLHY* and *MtPRR5* in the circadian clock term were identified as key targets (Figure 2E, Supplementary Materials Table S2), which is consistent with a recent report that proves the role of *MtLHY* in nyctinastic leaf movement [23]. In biological regulation terms, the expression level of three phytochrome kinase substrate genes were found to be significantly altered (Figure 2E, Supplementary Materials Table S2). In addition, we found nine potassium channel and three chloride channel-related genes differentially expressed in transmembrane transport term (Figure 2E, Supplementary Materials Table S2). Taken together, these DEGs provided valuable candidates to test their roles on the signal transduction of biological clock, light and ion channel during the process of motor cell turgor changes [2,3,8].

Apart from these DEGs consistent with the previous model, we identified several new biological processes. In the plant hormone signal transduction pathway of KEGG analysis, we found 77 auxin-related genes with the down-regulated expression in the *elp1-1* mutant, including 61 SAUR family genes, 8 AUX/IAA genes, 3 GH3 genes, 3 ARF genes, 1 TIR gene and 1 YUCCA gene, indicating the strong relationship between pulvinus development and the auxin signal pathway (Figure 2E, Supplementary Materials Table S2). At the same time, 75 DEGs were mapped into cell wall organization or biogenesis terms, including genes for xyloglucan endotransglucosylase/hydrolase family proteins, expansin and pectin methylesterase (Figure 2E, Supplementary Materials Table S1). It strongly suggests that the cell wall structure plays a key role in the function of pulvinus. In photosynthesis terms, there were 33 up-regulated genes mainly for photosystem I/ II reaction center-related proteins located in the chloroplast, implicating changes in photosynthetic activity of the pulvinus (Figure 2E, Supplementary Materials Table S2). Interestingly, we found 11 genes significantly regulated by ELP1 among the 47 novel genes, with 9 down-regulated and 2 up-regulated in *elp1-1* mutants compared with WT (Supplementary Materials Figure S3, Supplementary Materials Table S5). Taken together, our transcriptome analysis suggests a substantial number of DEGs are involved in multiple layers of gene regulation in pulvinus development.

To facilitate future study, it is necessary to know the distribution of the ELP1 binding cis-elements in these 2652 DEGs. Based on previous study, we found that LBD protein could recognize ‘GCGGCG’, ‘CGGCG’ or ‘GCGGC’ consensus sequences as direct binding sites [24,25]. After we analyzed the promoter regions of the 2652 DEGs, there are 592 and 878 genes that had an LBD binding motif from 1146 up-regulated and 1506 down-regulated DEGs, respectively (Supplementary Materials Figure S4). 

### 2.3. Multiple DEPs Are Involved in Pulvinus Development

**Figure 3:**
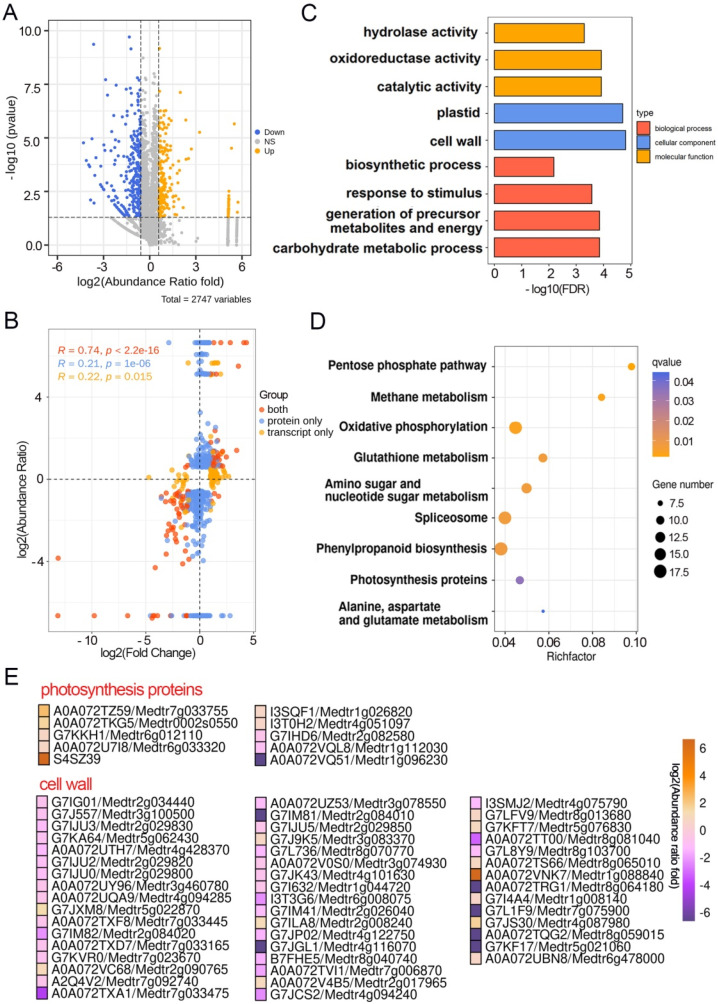
Multiple DEPs are involved in pulvinus development. (**A**) Volcano plots of differentially expressed proteins of WT and *elp1* mutants after proteomic analysis. (**B**) Comparison of expression ratios from transcriptomic (*x*-axis) and proteomic (*y*-axis) profiling. Log_2_ expression ratios were calculated from *elp1* mutants vs. WT. Significant expression changes were labeled in colors: blue points, proteins only; orange points, transcripts only; red points, both. Concordance tests revealed that *r*_Pearson_ = 0.74 between mRNA and protein ratios. (**C**) Gene ontology (GO) enrichment analysis of the DEPs between WT and *elp1* mutants. (**D**) Pathway enrichment of differentially expressed proteins. (**E**) Selected significantly differentially expressed proteins in photosynthesis proteins and cell wall.

Transcriptome and quantitative proteomes data provided an alternative to make a direct comparison between transcript and protein in the process of pulvinus development. By quantitative proteomic analysis, 2747 different proteins were detected, which covered approximately 5.4% of Mt4.0 annotation genes [21]. To ensure the accuracy and reliability of this experiment, differentially expressed proteins (DEPs) were identified with restrictive conditions of │Log_2_ (abundance ratio fold)│ ≥ 0.58 and *p*-value < 0.05. Based on these criteria, there were 621 DEPs including 251 up-regulated and 370 down-regulated in the *elp1-1* mutant compared to the wild type (Figure 3A, Supplementary Materials Table S6). Moreover, expression changes plotting against all the transcripts and proteins indicated that there was a moderate correlation (*r* = 0.74, *r* represents the Pearson correlation coefficient) (Figure 3B). Considering only significant changes, we found 118 genes specifically in the transcriptome and 525 proteins uniquely in the proteome to be differently regulated, while 94 DEGs/DEPs were detected in both the transcriptome and proteome data (Figure 3B, Supplementary Materials Table S7).

GO analysis indicated that DEPs were enriched in different terms, including carbohydrate metabolic process, plastid, cell wall, hydrolase activity and oxidoreductase activity (Figure 3C, Supplementary Materials Table S8). KEGG analysis revealed that the DEPs were enriched in photosynthesis proteins (Figure 3D, Supplementary Materials Table S9). For comparison, both cell wall and photosynthesis terms were enriched in DEPs and DEGs (Figure 2C–E and Figure 3C–E). In addition, there were 94 overlapping DEGs/DEPs, which comprised cell wall-related genes including glycosyl hydrolase and expansin (Supplementary Materials Tables S7 and S10). Taken together, proteomics analysis demonstrated both cell wall biosynthesis and photosynthesis-related proteins are involved in the pulvinus development, which is consistent with the transcriptome analysis.

### 2.4. Leaflet, Pulvinus and Petiole Tissue Display Different Gene Regulatory Landscape

**Figure 4:**
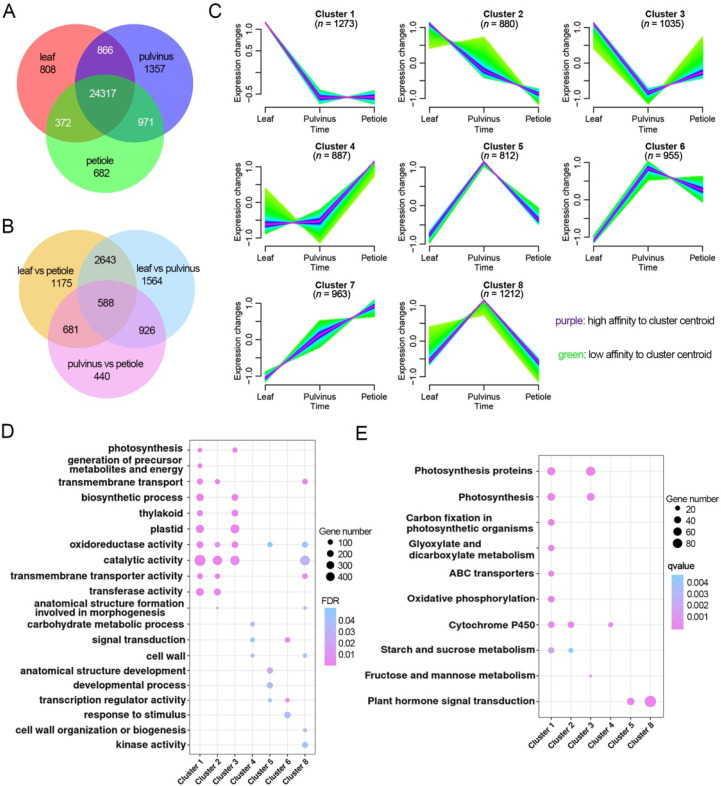
Different gene regulatory landscape of leaflet, pulvinus and petiole tissues. (**A**) The Venn diagram of the mRNA numbers detected in leaflets, pulvini and petioles, respectively. (**B**) The Venn diagram of the differentially expressed genes numbers by comparison among leaflets, pulvini and petioles, respectively. (**C**) Eight expression profile types of differentially expressed genes in (**B**) by fuzzy C-Means clustering. High affinity to the cluster centroid is shown in purple and low in green. (**D**,**E**) GO enrichment (**D**) and KEGG (**E**) analysis of each expression profile type in (**C**).

The leaf primordium initiates from the shoot apical meristem and subsequently goes through continuous processes of cells proliferation and differentiation. After undergoing the primary and secondary morphogenesis, the leaf primordium of *M. truncatula* develops into a compound leaf that consists of leaflets, pulvinus and petiole tissues. In order to address where the pulvinus comes from, we harvested these three types of tissues from 6-week-old seedlings and performed transcriptome analysis. The results revealed that a total of 26,363, 27,511, and 26,342 genes were detected in leaflet, pulvinus and petiole tissues, respectively (Figure 4A). Principle component analysis (PCA) and heatmap analysis of these transcriptional data exhibited apparent separation among these three types of samples, indicating their significant differences (Supplementary Materials Figure S5). With restrictive conditions of │Log_2_ FC│ ≥ 1 and *p-*adjust value < 0.05, there were 5721, 2635, and 5187 differentially expressed genes identified in the comparison between leaflet and pulvinus, petiole and pulvinus, and leaflet and petiole, respectively (Figure 4B, Supplementary Materials Tables S11–S13). Intriguingly, the lowest number of 2635 DEGs was found between petiole and pulvinus, suggesting that these two types of organs share the highest number of genes during their developmental processes.

Next, we use fuzzy C-Means to cluster these differentially expressed genes among pulvinus, leaflet and petiole into eight expression profiles. The analysis revealed that a large set of genes (*n* = 3188, clusters 1, 2, 3) was expressed at high levels in the leaflet (Figure 4C). These genes were significantly enriched in GO terms such as photosynthesis, biological process and transmembrane transporter activity. In KEGG analysis, these genes were enriched in photosynthesis, starch and sucrose metabolism terms, which are consistent with the function of leaflet as a photosynthesis organ (Figure 4D,E, Supplementary Materials Tables S14 and S15). Those genes with a high expression level in the petiole (*n* = 2805, clusters 4, 6, 7) did not display many GO and KEGG terms (Figure 4C–E, Supplementary Materials Table S14 and S15), while these genes’ high expression levels in the pulvinus (*n* = 2979, clusters 5, 6, 8) were significantly enriched for GO terms for ion binding, biological process, and KEGG terms for plant hormone signal transduction (Figure 4C–E, Supplementary Materials Tables S14 and S15). Taken together, these results provided a primary framework for understanding of the gene regulatory landscape responsible for the organ identity of pulvinus, leaflet and petiole development in compound leaves.

### 2.5. The Expression Level of Multiple Pulvinus Specific Expression Is Altered in elp1 Mutant

In cluster 5 and 8 (Figure 4C), we noticed that the expression of a number of pulvinus-specific genes, implicating their unique contribution to the pulvinus development in compound leaf. These findings prompted us to address what is the relationship between pulvinus-specific expression genes and 2652 DEGs in *elp1* mutant.

**Figure 5:**
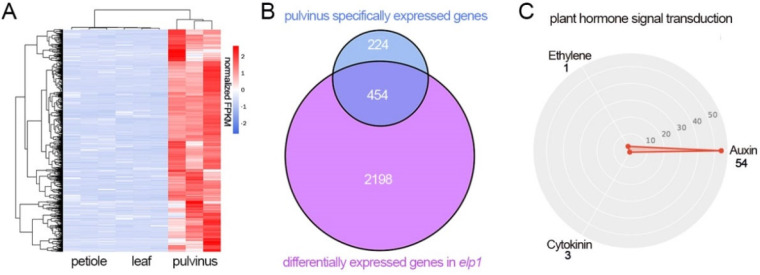
The expression level of multiple pulvinus-specific expression is altered in *elp1* mutant. (**A**) Hierarchical clustering of pulvinus specifically expressed genes in A17 leaflet, pulvinus and petiole. (**B**) The Venn diagram of comparison between the 678 pulvinus specifically expressed genes and 2652 DEGs in *elp1* mutant. (**C**) Classification of plant hormone signal transduction term.

In order to answer this question, firstly we identified 678 pulvinus-specific expression genes by the gene regulatory landscape analysis of leaflet, pulvinus and petiole tissues (Figure 5A, Supplementary Materials Table S16), and then compared these 678 specific expression genes with 2652 DEGs in the *elp1* mutant. This analysis helped us to identify 454 DEGs that were specially expressed in pulvinus, which covered approximately 17.1% (454/2652) of the DEGs and 67% (454/678) of the pulvinus-specific genes (Figure 5B). Thus, this reflects that most of the DEGs in the *elp1* mutant (2198/2652, 82.9%) were also expressed in the leaflet and petiole of the compound leaf, indicating that a lot of biological processes are shared between these three kinds of organs. On the other hand, the majority of pulvinus-specific genes were significantly regulated by ELP1, demonstrating the core role of ELP1 in the determination of pulvinus development and signal transduction. Noticeably, KEGG analysis found that a lot of auxin-related genes were enriched in the pulvinus (Figure 5C), suggesting that the auxin signaling pathway is associated with ELP1 function and pulvinus development. In conclusion, these results uncovered a more comprehensive gene regulation landscape and ELP1 function in pulvinus development.

### 2.6. The Auxin Is Involved in the ELP1 Regulated Pulvinus Development

**Figure 6:**
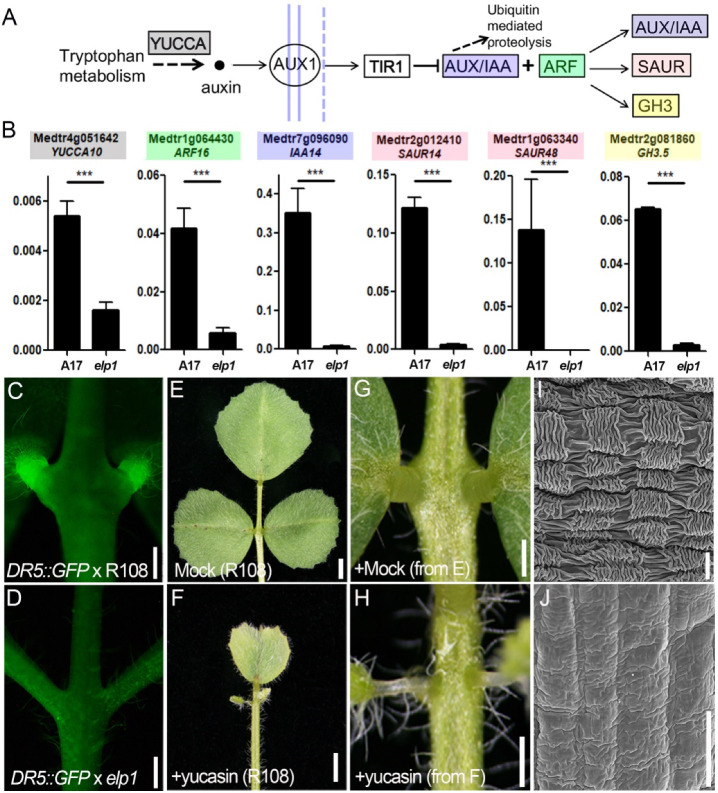
The auxin pathway is involved in the *ELP1* regulated pulvinus development. (**A**) Diagram of auxin biosynthesis and signaling pathway-related genes. The colored genes are corresponding to the genes listed in figure (**B**) with same color, respectively. (**B**) RT-qPCR of auxin related genes transcription in pulvinus of WT and the equivalent position of *elp1* mutants. Values shown in the form mean ± SD (*n* = 3), and asterisks indicate a statistically significant difference between WT and *elp1* (***, *p* < 0.001; *t*-test). (**C**,**D**) GFP signal in *DR5::GFP* transgenic lines of WT (**C**) and *elp1* mutants (**D**). GFP signals are significantly detected in pulvinus of WT but not in *elp1* mutants. (**E**,**F**) *M. truncatula* wild-type (R108) plants treated without (**E**) or with 250 µM yucasin (**F**), a specific chemical inhibitor of YUCCA protein, for 4 weeks were photographed. (**G**,**H**) Partially magnified view of pulvinus corresponding to (**D**,**E**), respectively. (**I**,**J**) Scanning electron microscope images of a pulvinus corresponding to (**G**,**H**), respectively. Scale bars: (**C**–**F**) 0.5 mm; (**G**,**H**) 2 mm, (**I**,**J**) 10 µm.

In this study, KEGG analysis of transcriptome data suggested that down-regulated DEGs in the *elp1-1* mutant are enriched in the plant hormone transduction pathway. It further revealed that most of members of this pathway were enriched in the auxin signaling pathway, in which 77 auxin-related genes, predominantly the SAUR, AUX/IAA and GH3 were significantly down-regulated in *elp1-1* mutant when compared to the wild-type control (Figure 2E, Supplementary Materials Table S2), indicating the requirement of a significant activation of the auxin signal transduction pathway for the normal pulvinus. RT-qPCR assay verified that the transcripts of auxin synthesis and response-related genes such as *YUCCA10*, *GH3*, *ARF*, *IAA* and *SAUR* were significantly down-regulated in the *elp1-1* mutant (Figure 6A,B). In addition, we monitored the activity of auxin response reporters *DR5::GFP* in WT and *elp1-3* mutants, and the results revealed that GFP signaling was only detected in pulvini of WT but not in the pulvini equivalent position of the *elp1-3* mutant, indicating that auxin accumulation and signaling could been tightly associated with the pulvinus development (Figure 6C,D).

To further dissect the relationship between pulvinus development and the auxin, we grew WT (R108) plants on MS medium with or without the supplement of yucasin, a chemical substrate that inhibits IAA production through blocking the YUCCA enzyme activity. The yucasin-treated plants displayed a malformed pulvinus phenotype after 4 weeks (Figure 6E–H). SEM images indicated that the yucasin-treated plants displayed a much smoother surface compared to the highly convoluted surface of epidermal cells of untreated plants (Figure 6I,J). In addition, the mutant of an auxin biosynthetic enzyme YUCCA1, *lls1*, also displayed malformed pulvinus phenotype (Supplementary Materials Figure S6), which further suggested that the auxin biosynthesis and its accumulation are very important for the pulvinus development.

### 2.7. Both the Cell Wall Compositiona and Chloroplast Enrichment Are Altered in elp1 Mutants

**Figure 7:**
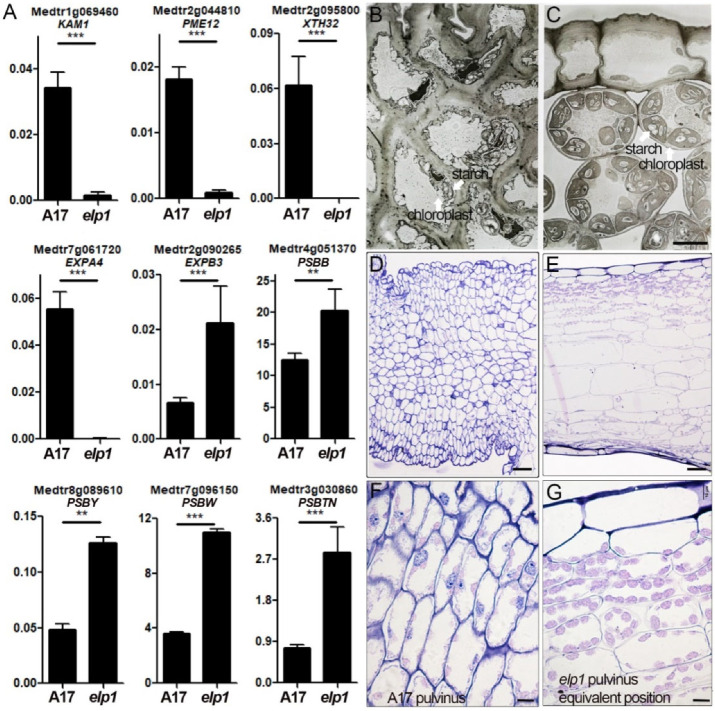
Both cell wall structure and chloroplasts distribution are altered in *elp1* mutants. (**A**) RT-qPCR analysis of cell wall and photosynthesis-related gene transcription in pulvinus of WT and the equivalent position of *elp1* mutants. Values shown in the form mean ± SD (*n* = 3), and asterisks indicate a statistically significant difference between WT and *elp1* mutants (**, *p* < 0.01; ***, *p* < 0.001; *t*-test). (**B**,**C**) Transmission electron microscopy images of a pulvinus of WT (**B**) and the equivalent position of *elp1* mutants (**C**), respectively. Cell arrangement and cell wall structure show the significant difference between WT and elp1 mutants. (**D**–**G**) Semi-thin sections analysis of a pulvinus of WT (**D**,**F**) and the equivalent position of *elp1* mutants (**E**,**G**). Scale bars, 5 μm in (**B**,**C**), 50 μm in (**D**,**E**), 10 μm in (**F**,**G**).

The motor organ pulvinus controls nyctinastic leaf movement through circadian rhythmic change in turgor and volume of motor cells. This allows us to assume that the unique structure of the pulvinus itself might play key functions during this kind of non-growth movement. Transcriptome and proteomic analysis have disclosed that cell wall and photosynthesis-related genes were significantly altered in *elp1-1* mutants, implying essential roles of cell wall structure and photosynthesis activity. RT-qPCR assay between WT pulvinus and *elp1-1* mutant verified the transcripts of cell wall and photosynthesis-related genes were significantly altered (Figure 7A). In addition, transmission electron microscopy studying indicated that motor cells of the pulvinus are connected to each other without free space by the thickened cell wall with very few but small chloroplasts (Figure 7B). By contrast, cells in the pulvinus-equivalent position of *elp1-1* mutants display elliptical and loosely arranged structure with large amounts of chloroplasts containing apparent starch article granules, and there is lots of intercellular space between them (Figure 7C). Similarly, cell morphology analysis of semi-thin sections indicated densely arranged motor cells with very few chloroplasts in wild-type pulvinus, while loosely arranged cells with chloroplast organelles are displayed in the *elp1-1* mutant (Figure 7D–G), implying the significantly different photosynthesis activity between WT pulvinus and the *elp1-1* mutant. Taken together, our results uncover a comprehensive transcriptome and proteome landscape that integrates hormonal and cell differentiation pathways to ELP1 function in acquiring the unique pulvinus structure. These anatomical and histological peculiarities, especially the chloroplast enrichment and motor cells arrangement, highlight the central role of ELP1 in the regulation of pulvinus development in *M. truncatula*.

## 3. Discussion

### 3.1. Comparison Analysis of Multi-Omics Data

**Figure 8:**
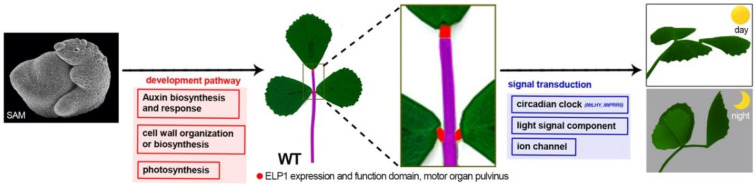
Schematic model for gene regulatory landscape of pulvinus development and signal transduction. The compound leaf consists of leaflets, pulvinus and petiole, and they initiate from the shoot apical meristem. During the pulvinus development process, the auxin biosynthesis and signal, cell wall and chloroplast-related genes are involved. At latter mature pulvinus stage, the circadian clock, light signal and ion channel-related genes participate in nyctinastic leaf movement regulation.

The phenomenon of nyctinastic leaf movement in *Fabaceae* and *Oxalidaceae* species has attracted scientists’ interest since the Darwin’s era [1]. However, the lack of public omics data on this special motor organ had limited our comprehensive understanding of the pulvinus development program and the signal transduction pathway underlying leaf movement. Here, combinations of omics analyses and other experimental data gave us a deeper understanding of pulvinus-driven leaf movement and the function of ELP1 (Figure 8). In addition, our data combined with previous microarray data of the abscission zone would provide further insight to decipher the mechanisms of leaf movement and leaf abscission.

### 3.2. The Signal Transduction and Developmental Pathway Are Altered in elp1 Mutant

Previous physiological research found that potassium channels, chloride channels as well as circadian clock and light signaling factors play vital roles in the regulation of osmotic pressure of motor cells during the leaf movement [3,5,7,11]. Moreover, *MtLHY* was proved to participate in leaf movement, and six potassium channel and three chloride channel-related genes and circadian clock-related gene *MtPRR5* were also found to be significantly regulated in the datasets (Figure 2E), suggesting their possible roles during the leaf movement. Recently, the rice clock component OsPRR73 was found to modulate OsHKT2; 1-mediated sodium homeostasis in response to salt stress, demonstrating the existence of direct interaction between clock factors and membrane channel genes [26]. In addition, three phytochrome kinase substrate (PKS) protein-related genes were found to be downregulated in *elp1* mutants (Figure 2E). PKS1 was identified as a substrate phosphorylated by phytochrome A that negatively regulated phytochrome signaling in *Arabidopsis* [27], and it was a likely candidate to participate in the signal transduction of leaf movement.

Auxin is one of the most important phytohormones, and it is also involved in most plant developmental processes, which tailors plant growth and morphology to environmental conditions [28,29]. In *M. truncatula, SLM1/MtPIN1*, *LOL1/AGO7*, *PHAN*, *MtREV1* and *MtYUCCA1/LLS1* were found to associate with auxin to affect leaf development [30,31,32,33,34]. Our results including transcriptional analysis, together with the phenotype analyses of IAA production inhibitor yucasin-treated plants, verified the essential role of auxin in pulvinus development (Figure 2D,E and Figure 6E–J). Further histological and morphological analyses of *lls1* found that extremely defective mutants of *lls1* display miniscule volume and abolish the central vascular bundle and more smooth epidermal cell appearance (Supplementary Materials Figure S6I–P). In conclusion, auxin biosynthesis and signal transduction are involved in the pulvinus development.

Both transcriptional and proteomics analyses indicated the alteration of a large number of genes expression and proteins enrichment of cell wall and photosynthesis, including xyloglucan endotransglucosylase/hydrolase, expansin, pectin methylesterase and photosystem I/II reaction center-related proteins (Figure 2E and Figure 3E, Supplementary Materials Table S2). Anatomical and morphological studies implied that motor cells display a thickened cell wall and significantly decreased enrichment of chloroplasts, which is consistent with the reduction in photosystem I/II reaction center-related proteins (Figure 7B–G). All these results suggest that ELP1 deploys gene expression in key processes, including auxin signaling, cell wall biosynthesis and chloroplast photosynthesis-related genes to sculpt and shape pulvinus into an efficient motor organ.

### 3.3. ELP1 Plays a Key Role in the Development of Pulvinus

In our comparative transcriptome analysis, the petiole and pulvinus tissues share most of genes with the least number of 2635 DEGs (Figure 4B), suggesting the possibility that petiole and pulvinus may initiate from the common primordia during the compound leaf development process. At the same time, previous research has suggested that the *elp1*/*plp* display a petiole-like pulvinus [13,14], and the histological and morphological analyses showed a similar appearance between petiole-like pulvinus and petiole tissue (Figure 1E–H). To distinguish these two types of tissues and make a better understanding of ELP1 function at the molecular level, we conducted a comparative transcriptome analysis between the 2652 (*elp1*/WT pulvinus) and 2635 (petiole/pulvinus in WT) DEGs. Results found a majority of DEGs were not overlapped, suggesting a significant difference between petiole-like pulvinus and petiole (Supplementary Materials Figure S7). Meanwhile, a large number of auxin, cell wall and photosynthesis-related DEGs were found in the 990 DEGs (Supplementary Materials Figure S7), suggesting the existence of a similar gene regulation pattern between petiole-like pulvinus and petiole. These results implied that the mutant of ELP1 led to the pulvinus exhibiting a petiole-like pulvinus morphology, which somewhat resembles but is significantly different from pulvinus and petiole tissue.

The expression level of most pulvinus-specific genes was significantly altered in the *elp1* mutant (Figure 5B), further demonstrating ELP1 function as a core regulator to control the gene expression level in motor organ, which is consistent with the phenotype of ELP1 as a determinant of pulvinus development. Importantly, further study of the 678 pulvinus-specific genes will make it possible to uncover the framework of the gene regulatory pathway and biological processes responsible for leaf movement. Recently, ELP1/PLP was identified as the master determinant in regulating the abscission zone development of leaflet and petiole, and loss of function of PLP leads to the absence of abscission zone (AZ) (Supplementary Materials Figure S8) [35]. The question of how ELP1 accurately regulates the development of pulvinus and its adjacent tissue abscission zone at the same time, so as to control leaf movement and abscission, is an interesting but applied topic for further study. Thus, it will be important to focus on dissecting the functions of DEGs in the *elp1* mutant, especially the pulvinus and abscission zone-specific DEGs. On that note, it will also be interesting to explore whether ELP1 and its homologs control a conserved gene regulatory network in determining pulvinus identity in Fabaceae, Oxalidaceae and Marantaceae species.

## 4. Materials and Methods

### 4.1. Plant Materials and Growth Conditions

The wild-type and *elp1-1* allele mutants for RT-qPCR, transcriptome and proteomic analysis are the A17 ecotype, while materials of *elp1-3* (NF2623), *lls1* for semi-thin sections, TEM and phenotype observations are R108 ecotype. The *elp1-1* allele was reported by our previous study [13]. In detail, a single nucleotide inserts between bases 53 and 54 of the ORF in the *elp1-1* allele, which would result in a frameshift and premature termination of the encoded protein. GFP signal analysis of *DR5::GFP*xR108 transgenic plants by using an Olympus BX63 fluorescence microscope were generated through introduction of the *DR5::GFP* plastid to R108 plants, while *DR5::GFP*x*elp1* transgenic plants were generated from hybrid progenies by crossing the *DR5::GFP*xR108 marker line with *elp1-3* mutants. *M. truncatula* plants were grown in a glasshouse with 15 h light: 9 h dark lighting conditions at 23 °C and c. 70% humidity.

### 4.2. RNA Extraction and Quantitative Real-Time PCR (RT-qPCR)

Nearly mature pulvini of WT plants and the pulvinus-equivalent position in *elp1* mutants were collected and ground to fine powder in liquid nitrogen. Total RNA was isolated with TRIzol (Sangon Biotech, Shanghai, China), and next the quantity and quality of RNA were assessed with Nanodrop 2000 (Thermo Scientific, Waltham, MA, USA), then approximately 2 µg of total RNA was reverse transcribed via HiScript Ⅲ 1st Strand cDNA Synthesis Kit (+gDNA wiper) (Vazyme, R312-01). cDNA corresponding to 25–50 ng of RNA was used as a template for RT-qPCR. RT-qPCR assays were performed with TsingKe Master qPCR Mix (SYBR Green Ⅰ) kit (TsingKe; 95 °C, 1 min; 95 °C, 10 s, 60 °C, 30 s, 40 cycles) by LightCycler 480 engine (Roche, Basel, Switzerland). The MtActin served as internal control and the primers in this study are listed in Supplementary Materials Table S17.

### 4.3. RNA Sequencing Assay and Data Analysis

Total RNA was extracted from pulvini, leaflet and petiole tissues of WT and the equivalent position of *elp1* mutants using the QIAGEN RNA prep plant kit. RNA quality was assessed on an Agilent 2100 Bioanalyzer (Agilent Technologies, Palo Alto, CA, USA). The cDNA libraries were constructed via the NEBNext^®^ Ultra™ RNA Library Prep Kit for Illumina^®^ (New England Biolabs, Ipswich, MA, USA) and then sequenced using Illumina HiSeq2000. All sequencing reads were trimmed low-quality bases and adapters with Trimmomatic (v.0.38) (Bolger AM, Golm, Germany), and quality checked by FASTQC (v.0.11.8) (Andrews S, Los Angeles, CA, USA). The paired-end cleaned reads were mapped to the *Medicago truncatula* reference genome (JCVI.Medtr.v4) using HISAT2 software (version 2.1.0) (hisat2 2.1.0 software, Dallas, TX, USA) with guidance by the corresponding annotations according to the parameters: max-intronlen = 4000, --rna-strandness RF and others were default parameters, and the transcription was assembled by Stringtie (1.3.4d) (Mihaela Pertea, Baltimore, MD, USA) [36]. Fragments per kilobase of exon model per million mapped reads (FPKM) for genes in each sample were extracted from the results of Stringtie, then scaled by a perl script with the upper-quartile normalization method. The differentially expressed genes were identified using DESeq2 [37] with |log2 fold change| ≥1 and *p*-adjust < 0.05. Expression profile types of the differentially expressed genes were then assigned by clustering using the Mfuzz program [38] and tissue-specific genes were calculated by TissueEnrich package [39].

### 4.4. New Gene Identification and Conservation Analysis

The new genes were identified according to three criteria as below: FPKM ≥ 2 in at least one sample, the transcript had complete CDS predicted by TransDecoder (Brian J Haas, Cambridge, MA, USA) and the length of encoded amino acid was more than 100 aa, and the new gene could be predicted by GeneMark-ET (Alexandre, Atlanta, GA, USA) [40], AUGUSTUS (version 3.4) (Katharina, Greifswald, Germany) [41] or GlimmerHMM (version 3.0.4) (W. H. Majoros, Rockville, MD, USA) [42] by inputting the gene and its flanking sequence (five reference genes on the upstream and the downstream, respectively). In addition, the new genes, which were in pairwise synteny with reference genes (MtrunA17r5.0) by genome synteny analysis, were regarded as equivalent genes and further removed. Amino acid sequences of new genes were compared to *Arabidopsis thaliana* (TAIR10), *Solanum lycopersicum* (ITAG3.2), *Oryza sativa* (RGAP7.0), *Glycine max* (Wm82.a2.v1), and *Cicer arietinum* (v1.0) by BLASTp with identity > 75% and coverage ratio > 70%.

### 4.5. Differential Protein Expression Analysis

Frozen WT pulvini and *elp1* mutant petiole-like pulvinus samples were ground with liquid nitrogen in a mortar. A total of 1 g of ground plant tissue was lysed in 6 mL protein extraction buffer (4% SDS, 100 mM DTT, 100 mM Tris-HCl, pH 8.5) and incubated at 95 °C under strong agitation for 10 min. After being sonicated at room temperature for 1 min, lysates were centrifuged at 16,000× *g* at room temperature for 10 min. Proteins were precipitated by adding two volumes of acetone to the supernatant. The pellets were washed three times with 70% acetone and dissolved in reducing buffer (1% SDS, 10 mM DTT, 100 mM Tris-HCl, pH 8.5). Proteins were digested by the Filter Aided Sample Preparation (FASP) method [43] in a 10k Da filter tube (Pall life sciences, Ann Arbor, MI, USA). Briefly, the reducing buffer was replaced by 8 M urea buffer; 50 mM ammonium bicarbonate was used to replace the urea buffer. Trypsin with a final 1:50 (*w*/*w*) ratio was added to digest proteins at 37 °C overnight. The digests were collected from the filter tubes and desalted using a Sep-Pak C18 column.

For dimethyl labeling, the tryptic peptides were dissolved in 100 μL 100 mM triethylammonium bicarbonate (TEAB) buffer (pH 8.5), and then 4 μL of ^13^CD2O (heavy) and CH_2_O (light) were added into the peptide sample of wild-type and *elp1* mutant, respectively. After mixing with 4 μL of fresh prepared 0.6 M sodium cyanoborohydride, the samples were agitated for 60 min at 25 °C. The reaction was stopped by adding 16 μL of 1% ammonium hydroxide. The samples were acidified by 20 μL of 10% formic acid. The light-labeled and heavy-labeled peptides were combined and desalted using a Sep-Pak C18 column.

Peptides were analyzed by nanoAcquity ultra performance LC (Waters, Milford, MA, USA) and Orbitrap Fusion mass spectrometer (Thermo Fisher Scientific, Watham, MA, USA). The peptides were loaded on 20 mm C18 trap column (180 μm i.d.) at the flow rate of 3 μL/min for 5 min and separated on a 100 mm C18 analytical column (1.7 μm particles, 100 μm i.d.) at 350 nL/min. Solvent A (0.1% FA) and solvent B (ACN/0.1% FA) were used as the mobile phases in the LC. Peptides were eluted using the mobile phases through a linear gradient from 5 to 35%, and then 50% of solvent B at a duration of 165 min. The instrument of a mass spectrometer was run in the data-dependent mode with dynamic exclusion. MS survey scan was performed by Orbitrap at a resolution of 120,000 over the m/z range of 350–2000, and precursor ions were selected for MS/MS measurements by high-energy collisional dissociation (HCD) fragmentation on Top Speed mode. The target values of automatic gain controls (AGC) were set up to 200,000 for Orbitrap MS and 50,000 for Orbitrap MS/MS detection. Dynamic exclusion was enabled for 60 s.

The raw files were searched against the uniprot *Medicago truncatula* database (57,065 sequences) using the Proteome Discoverer 2.2 software (Thermo Fisher Scientific, Watham, MA, USA). Dimethyl-labeling quantitation was performed using the software with the default parameters. The search parameter for tryptic digestion was restricted to two missed cleavages of proteins. Carbamidomethylation of Cys was designated as a fixed modification. Light dimethyl label of Lys or peptide N termini, heavy dimethyl label of Lys or peptide N termini, and oxidation of Met were considered as variable modifications. The precursor mass tolerance was set up to 10 ppm, and MS/MS fragment tolerance was set to 0.1 Da. The false discovery rate (FDR) of protein and peptide assignment was set up to less than 1%. In order to remove systematic bias, a normalization mode for total peptide amount was performed by the Proteome Discoverer 2.2 software before the quantitative analysis. This calculates the total sum of the abundance values for each channel of all peptides identified within a file. It then takes the channel with the highest total abundance as a reference and corrects all abundance values in all other channels by a constant factor per channel, so that at the end the total abundance is the same for all channels. In this study, proteins were considered to be significant if they had a *p*-value < 0.05 using the *t*-test and |log2 (abundance ratio fold)| ≥ 0.58.

### 4.6. Functional Annotations and Enrichments

Functional genes and proteins were annotated including gene ontology (GO) obtained from InterProScan (v5.44) [44] and EggNOG-mapper (v2) database [45], and KEGG orthology (KO) acquired by KAAS web server (KAAS: an automatic genome annotation and pathway reconstruction server. Available online: https://www.genome.jp/tools/kaas/ accessed on 3 April 2021) and EggNOG-mapper (v2) database [45]. Then, the python library named GOATOOLS [46] was used for the enrichment of differential expression genes and proteins with FDR error-corrected *p*-value < 0.05, and KEGG enrichment analysis was finished under an R package named clusterProfiler [47] with q-value < 0.05.

### 4.7. Paraffin Sections

Mature pulvini from 8-week-old plants were collected and fixed in FAA fixation solution and subsequently dehydrated in a graded ethanol series (50%, 60%, 70%, 80%, 90%, 100%), each grade for 2 h, then the materials were submerged in 50% ethanol and 50% xylene for 2 h, subsequentially in xylene for 2 h, and then we gradually added the paraffin wax to the xylene for 2 d at 60 °C. Next, embedding the materials in paraffin wax was conducted and they were then cut at 10 µm. After mounting sections onto slides and baking in 42 °C for 6 h, we then deparaffinized sections in two changes of xylene for 10 min each, hydrating the sections in a graded ethanol series (100%, 90%, 80%, 70%, 60%, 50%, 30%), each grade for 5 min. Next, toluidine blue staining for 30 s was conducted, followed by rinsing in distilled water. All sections were mounted with microscope (Olympus BX63, Tokyo, Japan).

### 4.8. Semi-Thin Sections

Mature pulvini from 6-week-old plants were collected and fixed in FAA fixation solution and subsequently dehydrated in a graded ethanol series (50%, 60%, 70%, 80%, 90%, 100%), each grade for 2 h, and then they were submerged in isopropanol and n-butyl alcohol for 6 h, respectively. After infiltration and embedding in Glycol methacrylate (GMA) for 2 weeks, semi-thin cross sections (2 µm) were cut by ultramicrotome (Leica EM UC7) and reacted with periodic acid-Schiff solution. All sections were mounted with a Olympus BX63 microscope.

### 4.9. Scanning Electron Microscopy (SEM) Analysis

Mature pulvini from 6-week-old plants were collected and fixed in 3% glutaraldehyde fixation solution and subsequently dehydrated in a graded ethanol series (45%, 50%, 60%, 70%, 80%, 90%, 100%), each grade for 0.5 h. SEM was carried out as described previously [48].

### 4.10. Transmission Electron Microscopy (TEM) Analysis

TEM assay was performed as previous protocol [49]. Briefly, pulvini were collected and embedded with Epon 812 (polymerization at 60 °C for around 48 h); next, by using a Leica EM UC7 ultramicrotome, ultrathin sections (60 nm) were prepared and loaded onto the 50-mesh Cu grids, and then double stained with 2% uranyl acetate and lead citrate. By using a JEM 1400 plus transmission electron microscope at 120 kV, sections were photographed.

### 4.11. Yucasin Treatment

Yucasin treatment assay was essentially carried out as previously described [34]. Briefly, R108 seeds were germinated for 3 d in 4 °C and then transferred into 1/2 MS media contain 250 µM yucasin. The seedlings were cultured under 15 h light: 9 h dark lighting conditions for 4 weeks, and the compound leaves were then detached for photographing.

## 5. Conclusions

In this study, the transcriptome and proteome analyses between WT pulvini and the *elp1* mutant elucidated that there are hundreds and thousands of mRNA expression and protein abundance with significant changes, which are probably related to pulvinus development and signal transduction. In addition, comparison analysis of pulvinus-specific gene expression in compound leaf provided a deeper understanding of the gene regulatory landscape and ELP1 function in pulvinus. Meanwhile, the auxin pathway, cell wall composition and chloroplast distribution were found altered in *elp1* mutants, verifying their involvement in the ELP1-regulated pulvinus development and the powerful value of these omics data. This study provides a rich resource to uncover the multiple layers of the gene regulatory network involved in pulvinus development and signal transduction in the model legume *M. truncatula*, further facilitating the investigation of ELP1 function on leaf movement.

## Data Availability

The raw data of the RNA-seq experiments have been deposited in the NCBI Sequence Read Archive (https://www.ncbi.nlm.nih.gov/sra accesced on 19 September 2021) under BioProject PRJNA764467, while the mass spectrometry proteomics data have been deposited to the ProteomeXchange Consortium (http://proteomecentral.proteomexchange.org accesced on 19 September 2021) via the iProX partner repository [50] with the dataset identifier PXD028750.

## Supplementary Materials

The following supporting information can be downloaded at: https://www.mdpi.com/article/10.3390/ijms23084439/s1.
