# Supplementary material for: Multidimensional Gene Regulatory Landscape of Motor Organ Pulvinus in the Model Legume Medicago truncatula
Source: Int J Mol Sci. 2022 Apr 18;23(8):4439. doi: 10.3390/ijms23084439 (PMC9031546; doi:10.3390/ijms23084439)
Supplement: Supplementary file 1 [file ijms-23-04439-s001.zip › Supplementary Files/Supplementary information.pdf]

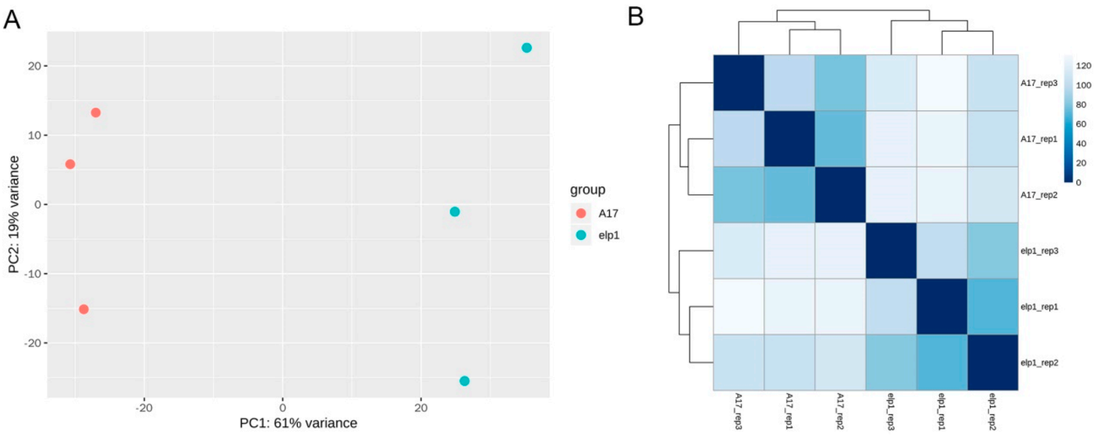

Figure S1 Principal component analysis (PCA) and heatmap analysis among three biological replicates from the WT (A17) and *elp1* mutant samples.

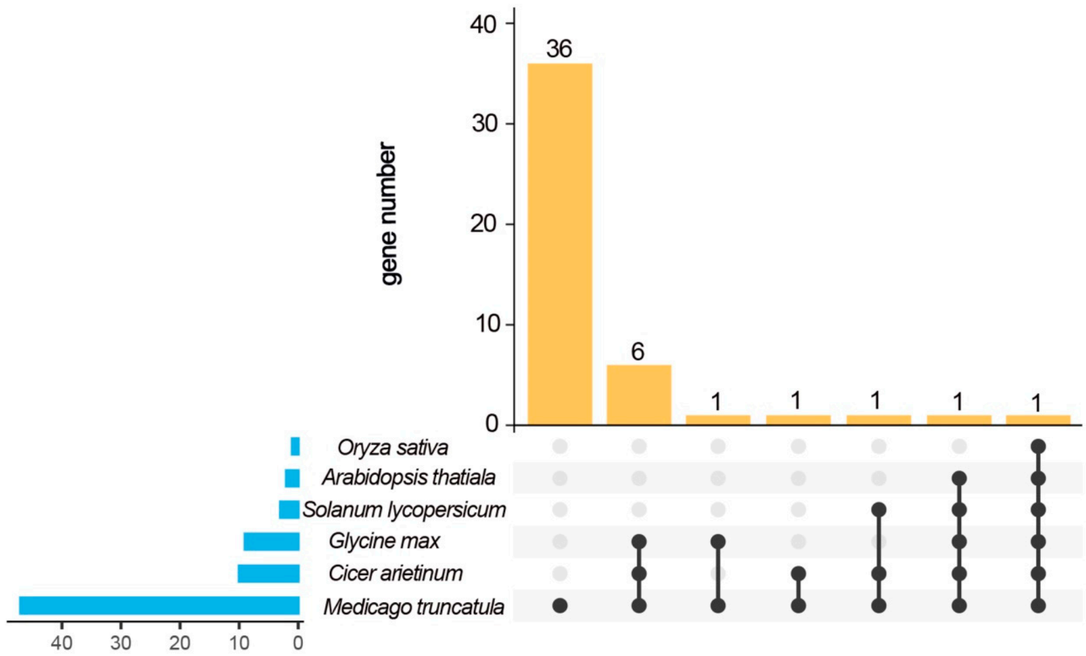

Figure S2 Upset plot comparing new genes identified in genome datasets of *Medicago truncatula*, *Cicer arietinum*, *Glycine max, Solanum lycopersicum, Arabidopsis thatiala* and *Oryza sativa* species.

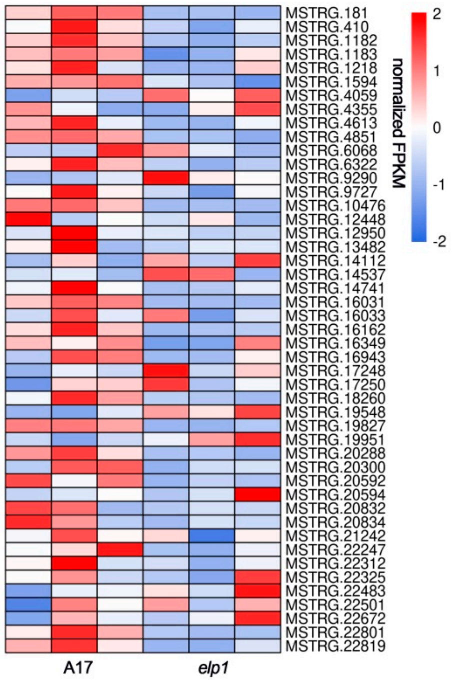

Figure S3 Heatmap analyses of 47 novel genes in WT (A17) and *elp1* mutant samples.

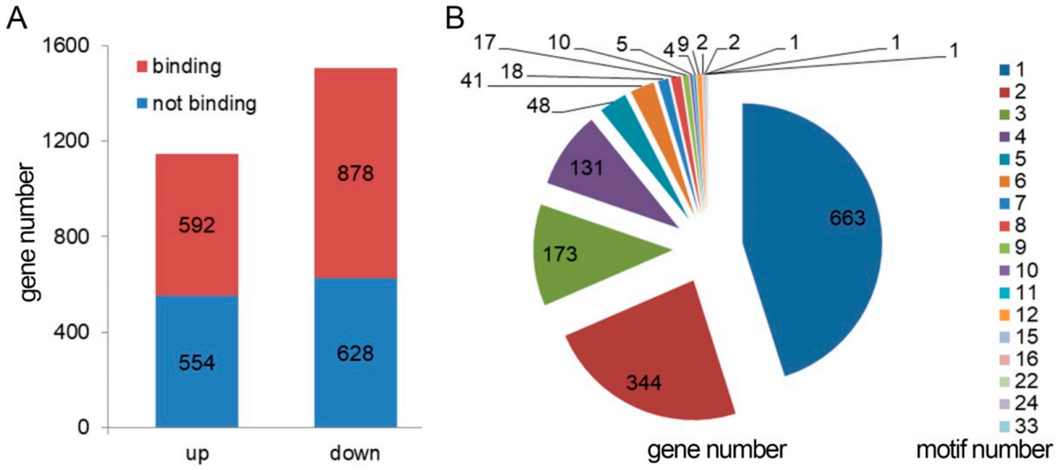

Figure S4 ELP1 binding targets analysis.
(**A**) ELP1 direct targets analysis from DEGs. Red, DEGs with ELP1 binding motif in the promoter region; blue, DEGs without ELP1 binding motif in the promoter region.
(**B**) Motif number statistics in the promoter region of ELP1 direct targets from DEGs.

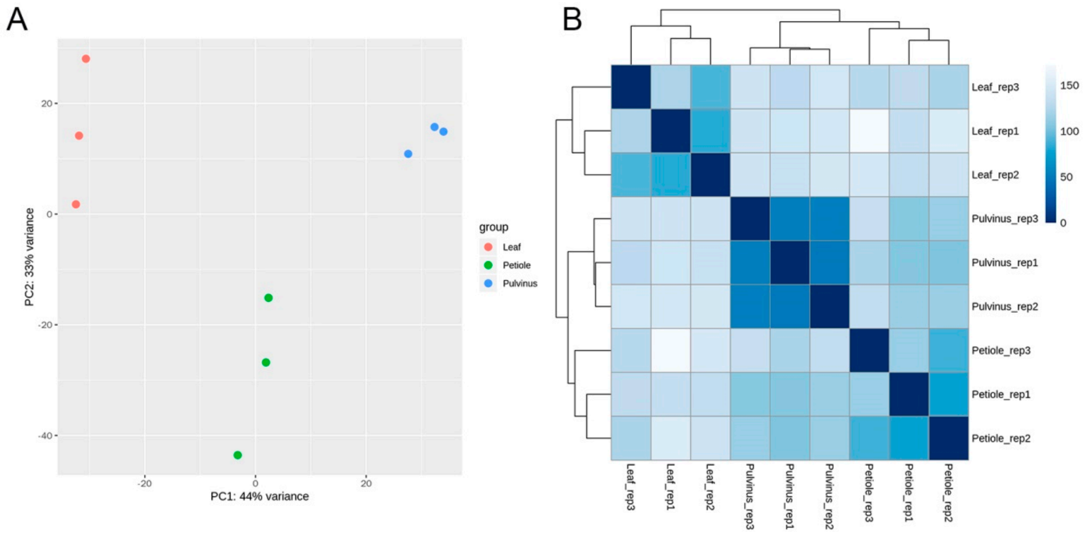

Figure S5 Principal component analysis (PCA) and heatmap analysis among three biological replicates from the petiole, pulvinus and leaf tissues.

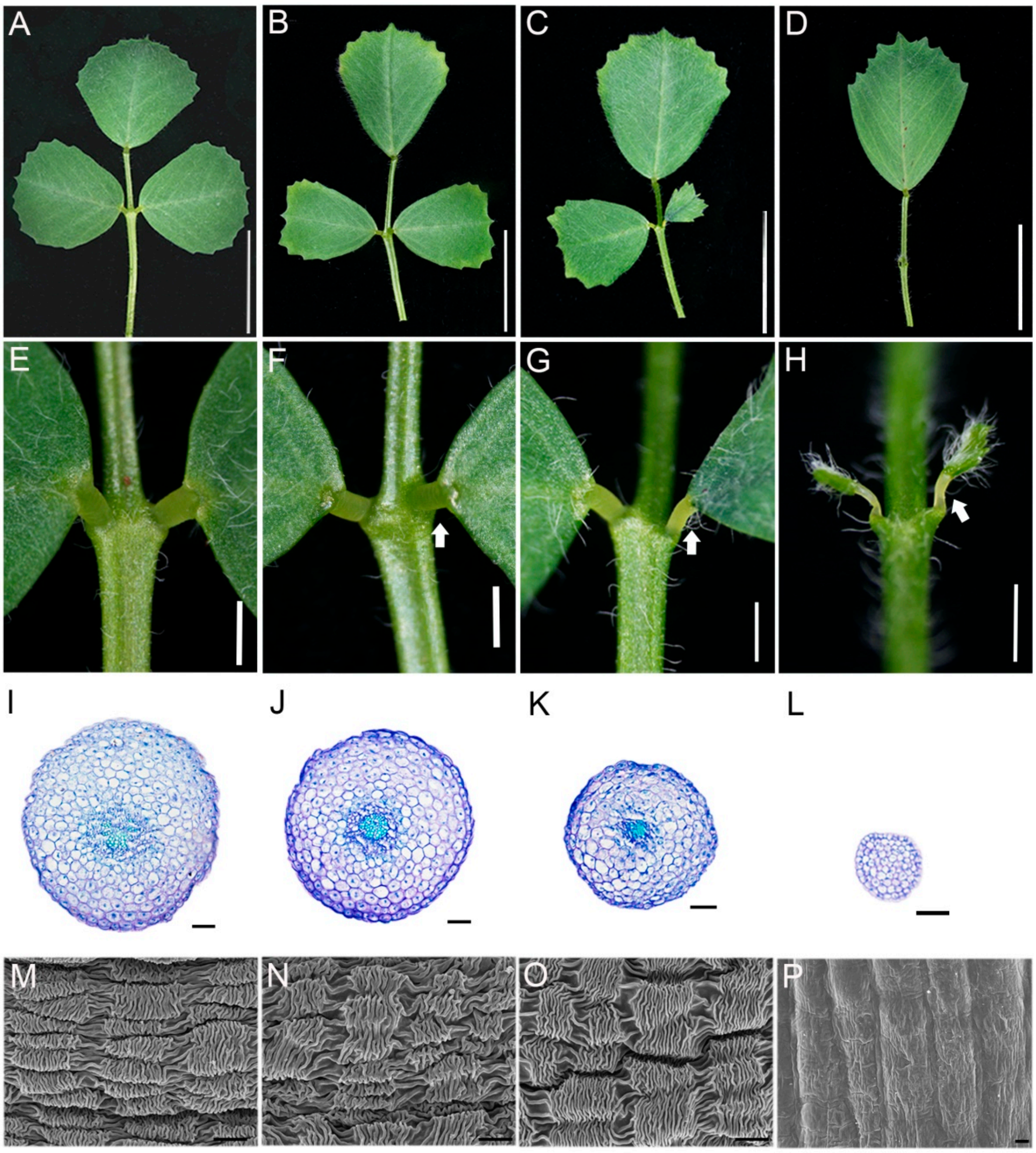

Figure S6 Pulvinus defect phenotype of *lls1* mutant.

(**A-D**) Morphogenesis of compound leaves of WT (**A**) and the moderately defective phenotype (B), severally defective mutant (**C**) and extremely defective phenotype (**D**) of *lls1* mutant, respectively.

(**E-H**) Morphogenesis of lateral pulvinus of WT (**E**), the moderately defective phenotype (**F**), severally defective phenotype (**G**) and extremely defective phenotype (**H**) of *lls1* mutant, respectively. Arrows indicate the lateral defective pulvini.

(**I-L**) Cross sections of pulvinus of WT (**I**), the moderately defective phenotype (**J**), severally defective phenotype (**K**) and extremely defective phenotype (**L**) of *lls1* mutant, respectively.

(**M-P**) Scanning electron microscope images of a pulvinus of WT (**M**) and the moderately defective phenotype (**N**), severally defective phenotype (**O**) and extremely defective phenotype (**P**) of *lls1* mutant, respectively.

Scale bars, 1 cm in (**A-D**), 1 mm in (**E-F**), 50 μm in (**I-L**), 10 μm in (**M-O**), 2 μm in (**P**).

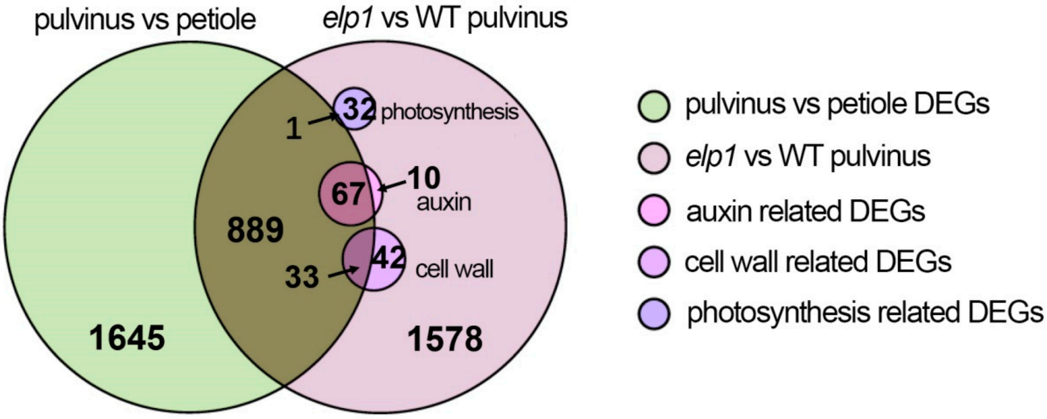

Figure S7 Comparative transcriptome analysis between the 2652 (*elp1* vs WT pulvinus) and 2635 (petiole vs pulvinus in WT) DEGs.

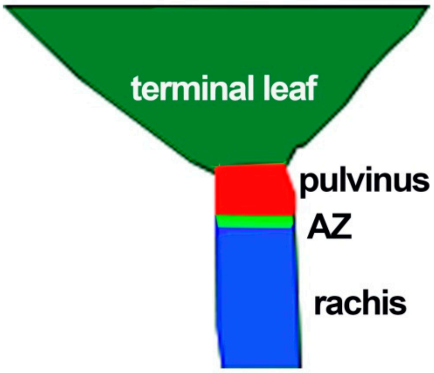

Figure S8 Schematic diagram for terminal leaf, pulvinus, abscission zone (AZ) and rachis position in *M. truncatula* compound leaf.
